# Abscess of the Brain

**Published:** 1908-09

**Authors:** R. C. Buckner

**Affiliations:** Ashville, N. C.


### ABSCESS OF THE BRAIN.

BY R. G. BUCKNER, ASHVILLE, N. C.

Abscess of the brain is a focal supurative inflammation of either or both the grey and white matter.

*Etiology.*—It is always secondary and dependent upon the intracranial invasion of micro-organisms from remote sources; any one of the pus-producing micro-organisms being sufficient cause. It may occur at any age, but it is more frequent in the second and third decenial, and is very rare in very young children and in old age. It is from three to five times as frequent in males as in females. It is associated, first, with local cranial supurations; second, with injuries to the head; third, with certain general infections; fourth, with certain local diseases in other parts of the body.

According to Newton Pitt, nearly half of all brain abscesses are associated with cranial suppuration, and nearly all of these are due to middle ear disease. Chronis otitis media is by far a more common cause than the acute form. Jansen's anylitical study of several thousand cases established the fact that the proportion is more than six to one; other observers have put it four to one. Other cranial suppurations sometimes follow by abscess in the causative relation are to be found in the frontal sinus, the ethnoid cells, the antrum, the orbit, and the nose and throat. Practically all cases occurring in very young children are due to trauma of middle ear disease.

L. E. Holt, in the Archives of Pediatrics, March, '08, gives the following study of thirty-two cases:

1. That abscess of the brain in children under five vears is rare; 2, that the principal causes are otitis and traumatism; 3, it rarely follows acute otitis, most often neglected cases, and is surely secondary to disease of the petrous bone; 4, in the case occurring in infancy without evident cause, the source of infection is probably the ear, even though there be no discharge; 5, the development of abscess after injury to the head without fracture of the skull is extremely rare. In nearly all the trauma cases definite cerebral symptoms show themselves within the first two weeks after the injury. In case of falls, as remote as several months, there is probably some other causes as a latent otitis.

Among the general infections most liable to become complicated with abscess of the brain are pyemia, tubercle, influenza, enteric fever, variola and erysipelas. Since pyemia is well on its way to becoming extinct, it is not so frequent a cause.

"It is of great interest that cases of abscess of the brain have been met with apart from any other macroscopic intracranial tubercular lesion which have yielded pure cultures of the tubercle bacillus."—Ballanoe.

Dr. Bristow, in 1891, published two cases following influenza without middle ear disease. Among local diseases in other parts of the body is putrid inflammation or gangrene of the lungs, suppurating cervicle glands and foci of suppuration of the liver, fallopian tubes, and at the seat wounds. An instance illustrating the last is reported by Surgeon General Turner, U. S. A., in the New York Medical Journal, March 14, 1891, of the sudden death of a soldier who was considered to be in perfect health, the autopsy showing a multiple abscess of the left frontal lobe; the man at the time of his death was reclining on a bench reading a newspaper. A few weeks previously, he had received a gun-shot flesh wound of the arm in an engagement with robbers, which had healed readily, the bone not being injured. The abscess could not be tracted to anything other than the injury of the arm, though there was not a single symptom mental or physical suggesting its presence.

Brain abscess occuring after gangrene of the lung has been observed and recorded at least fifty years, though it is evident that the infection is carried in the bloodstreams, no adequate explanation as to why it should be localized in the brain has been given. In 1901, Claytor collected reports of fifty-eight cases secondary to diseases of the lungs, nearly all of whch occurred in the left side of the brain. In twenty of the cases the lung disease was bronchiectasis; in ten empyena; in nine, purulent bronchitis; in seven, gangrene; in five, tuberculous disease; in three, abscess of lung; in two, pneumonia, and in two gun-shot wounds of the lung.

Stoll reports a case of abscess in left frontal lobe and a cavity in the apex of the right lung 2 1-2 inches in diameter.

Blotche found pulmonary pigment in the pus of a vertian brain abscess.

In reference to injuries, I will only state that except when the instrument causing the injury has penetrated deeply into the brain substance, the abscess is usually really a local meningial suppuration with participation of the adjacent brain cortex, a meningo-cortical abscess rather than a brain abscess proper. Occasionally injury leads to chronic disease of the bone from which a brain abscess may arise.

*Pathology of Infection.*—Infective processes may extend from a focus of cranial disease to the interior by a visible continuous track of diseased one, or through a foramen or canal for the passage of vessel or nerve, or along the processes of dura mata which in certain situations dip into the bone, or by entering the circulation. In some injuries infected material is introduced directly into the brain or the cranial cavity, as shown by a stab culture being made. This is the only difference in the pathology of intracranial infection in case of injury and disease. The infective process spreads more or less rapidly from the spot where the dura has been brought in contact with the infested material. Here the dura becomes inflamed and extra dural suppuration occurs. This is the first state of intracranial infection, and further extension may be prolonged on account of the great resistance of the dura, and upon this depends the extent of the localized extra dural abscess. The dura may be softened and perforated immediately with only a few drops of pus collecting between it and the bone. The resistance of the dura is illustrated by Bergmann's case. On April 2nd, a man was admitted to the hospital with middle ear disease. On irrigating of the ear until it was quite free from pus, the auditory canal was rapidly re-filled to overflowing, and a mastoid operation done on Harch 12th. The following day, when dressing and inspecting, a fistulus track was detected. This was enlarged with a sharp spoon. There was a recurrence of symptoms. On April 11th, a free opening was made by chisseling away considerable cone, thus freely opening the extra dural abscess. From that time recovery was uninterupted. Pus evidently had been in contact with the dura for probably more than nine weeks, but no perforation had taken place. When the infection traverses the arachnoid, and reaches the subarachmoid space and the pia, there will be either a localized or a diffused inflammation, the extent depending on the virulency of the infection.

The infection in disease of the cranial bone is by direct continuity in the continuous track of diseased bones. In a rapidly extending infective process, diffuse meningitis would be the most probable result; in the more slowly spreading infections resulting from chronic disease, the meningeal affection would be localized by adhesion, and time given for extension of disease of the brain. This is shown by the fact that abscess of the brain or sinus infection is more common complicaton of chronic ear disease than is acute suppurative meningitis. In most cases of slowly spreading infection from chronic disease, adhesions occur obliterating the arachnoid space at the site of infection, and binding together the dura, arachnoid, pia, and cortex. The lymphatic shields of the numerous small blood vessels which traverse the cortex at right angles to its surface, are in direct communication with the subarachnoid space, and through these as through a number of capileary tubes infective matter easily traverses the cortex and reaches the white substance. The cortex is very vascular, and its connective tissue element is reinforced by numerous folds of promulgation from the pia, and abundantly supplied with the connective tissue corpuscles, hence, it is able to offer a strenuous resistance to the bacterial attack, and does not usually undergo any extensive material, a barrier of fibrous tissue is formed, limiting the destructive process to the narrow track.

The white substance is much less resistant, because it is non-vascular, and the greater the distance from the cortex, the more easily does the bacterial action cause its dissolution. In its incipiency it presents the local appearance of what has been described as acute red softening; the degree of redness depending upon the amount of blood determining to the point. As it advances, the pus changes from a reddish-yellow to a greenish, or greenish-yellow color, and in some cases the odor is quite offensive. This abscess comes to assume a mushroom shape with the stem attached to the dura at the original site of infection from the bone. When the dura has been separated from the bone over a considerable area, there is a greater extent of adhesion of the meninges. Salzer successfully operated on a case where the tempero-phenoidal dura was separated from the bone over an area as large as a silver dollar, and was in a sloughy condition. The sloughing portion was excised and the meninges were diffused into one layer; the inner portion, formed by the pia, was not necrotic, and there was no abscess of the brain.

Mannasse reported a case in which the infection had proceeded further, and there was abscess of the brain, the outer wall over which a considerable area was formed by fused meninges and cortex. The more recent the abscess, the nearer it will lie to the spot where the infection traversed the dura, and the more evident will be the stalk, or its remains. The older the abscess the greater is the apparent recession from the dura, and the less evident the stalk. Thus the infection gradually spreads into the brain substances by slow extension in direct continuity from the spot where the disease in the bone reached the interior of skull. Yet infective particles may, in the brain, as in other parts of the body, be carried by circulation to a spot remote from the site of infection. An abscess may thus arise in the substance of the brain without any visible connection with the bone disease to which it really owes its origin. The stalked form of brain abscess compares in its mode of formation to palmar abscess, from which infection in an abrasion of the skin of the palm, being connected with the superficial area by a narrow tract to a focus of disease beneath the deep palmar fascia, and the isolated brain abscess has its parallel in an abscess of liver arising from disease of the intestine.

The explanation of the pathology is not far to see in a case reported by Swain, in which purulent infection of the choroid plexus in the descending cornu of the lateral ventricle occurred as a result of caries of the tegmen tympani of the same side, the intervening brain substance being unaffected. In rapid increase of the abscess it may lead either into the ventricles or on the surface of the brain.

Encapsulation of abscess in the brain is thought to be relatively more frequent than in other parts of the body. The pathological process is the same, but the peculiar liquid texture of the brain allows a sharper differentiation between the hard tissues forming its wall, and the surrounding unaltered brain substance. Acute brain abscesses are sometimes encapsulated, and nearly all chronic abscesses are encapsulated.

In a case of cerebellar abscess, with symptoms pointing to a duration of at least eight months, no capsule is found, but the whole cerebellar hemisphere was nothing but a shell of softened gray matter. As in other parts of the body, the latent abscesses may slowly extend and give rise to slight symptoms extending over a considerable period, and encapsuled. A slowly growing abscess may be regarded as pushing aside fibres passing from the cortex to the internal capsule, rather than destroying them. This inference is supported by the fact that recovery from paralysis takes place after successful drainage of the abscess, yet it should be remembered that cortical impulses may find new paths.

When an abscess is drained through the stalk, as in the case of tempero-sphenodal agscess opened through the tegmen, though it may be large, there may be but little actual damage to the cortex. A thick capsule does not prevent the abscess from extending, nor even from leaking into the ventricle. It is a fact also, that acute inflammatory softenings, or even suppurations, have been known to arise around an encapsuled abscess. Abscesses in thick capsules, which can be shelled out whole, have run a chronic course. Complete encapsulation of the stalk form does occur, the narrow track of communication being obliterated by scar tissue. In such cases the capsule was found to be adhered to the bone.

When an abscess is found in the brain completely isolated, and at some distance from the meninges, the infective organism has been carried by the blood or lymph stream, and has multiplied at a spot some distance from the original point of infection. Many such cases have resulted from injury without bone disease, a considerable number having followed gun-shot wounds. The complications usually found are phlebitis and thrombosis of the lateral and the superior petrosal, sinus leptomeningitis, extensive meningo-encephalitis, and purulent pachy-meningitis, leptomeningitis and sinus thrombosis being especially common in cases of aural disease.

Streptcocus pyogenes albus, staphlyoccus cereus flavus, and the bacterium vulgarius, Charcot and Leyden crystals and streptothrix, have been found in pus from cerebral abscess.

Spontaneous recovery in certain tubercular cases have been cleamed by competent observers. Inspissation, and even calcification of the brain abscess has been observed, but only in tuberculous cases. It is stated by Ballance that cases are relatively common in early life, which either get well or turn a chronic course, extending over many years, and then die from distention of the ventricles; and the only explanation of the symptoms can be given by inferring the presence of cerebral tumor, or of menengitis. In these cases it seems probable that there was a local tuberculous mass in the brain from which recovery had come. In one such case some four years after a diagnosis of cerebral tumor had been made, the autopsy showed great distension of the ventricle. There was no visible tumor, and no evident trace of tubercle in the brain. But in the mesentery there was a large calcareous mass.

Two girls under twenty years of age both suffering from headache, vertigo, mistagmus and repeated purposeless vomiting; both had double optic newritis, unsteady gair, and agsence of the patellar reflex. The diagnosis in both cases was some affectation below the tentorium, probably tumor; both made good recoveries, but in one, some impairment of sight remained.

Abscess of the brain is commonly single. MacEwen says 93 per cent. of abscesses from injury are single. In pyemia they are usually multiple. A second abscess occurs in the frontal and occipital lobe occasionally, and even more often in the tempero sphenoidel lobe.

*Symptoms.*—These may be broadly divided into general and focal symptoms. Among the former are headache, photophobia, slow cerebration, slow pulse, subnormal temperature, occasional chills, foul breath, deathly pallow of the skin, constipation, facial palsy of the peripheral type, optic neuritis, impaired vision, conjugate deviation of the eyes, stabile pupil, mystagmus. The mental state is very likely to be mistaken. In order to estimate exactly it is well to know the mental capacity of the patient. A condition of excitement and talkativeness in a person who is reserved and stolid, is of much more importance than in an excitable and loquacious individual. Also apathy an dsupor in a man of bright active intelligence is of greater diagnostic value than in one who is dull and stupid. These variations in the mental state are observed also in a sinus thrombosis, meningitis and tumor.

Headache is a variable symptom, there being few cases recorded in which it was never noted, and other cases in which it was not a very marked symptom until late in the disease, and still other cases in which it occurred at different times, but was never severe, and still other cases in which it was the chief symptom.

The course of the temperature is also quite variable. MacEwen points out that it is common in abscess of the brain to find a persistently low temperature with little variation, and that during the preliminary period it is usually slightly above normal. During the period of full development of the abscess, it is about normal, or slightly subnormal, from 97 to 99 F., and in the termineal stage if the abscess burst the temperature rises within a few hours in a bound to 105 F., but if it is evacuated by operation, it rises to about 101 F., and in a few hours falls below 100 F., remaining around normal until recovery. Okada found a marked rise of temperature and febrile course in forty-six out of eighty-eight cases of abscess of cerrebellum. In fifteen the temperature was normal, and in fifteen it was subnormal. In eight there was a rise of temperature only at the onset, and in four only at the very end.

Rapid emaciation, the cachectic appearance, with sallow skin and evidences of the septic state, and other above described symptoms, suggest latent abscess.

Optic neuritis is a valuable sign when taken with other symptoms. In Okada_s cases, two-thirds of his patients had eptic neuritis in one or both eyes. In groups reported by other observers, it occurs in 30 per cent. of the cases, but it occurs also in brain tumors, meningitis and sinus thrombosis.

The following localizing symptoms should be taken into account:( Motor aphasia, optical aphasia, hemianopsia, hemianesthisia, and weakness of the arm and leg, vertigo, ataxia, with purposeless vomiting, lying prone on the side of the lesion, or falling toward the side of lesion, etc.

Careful observation and record of the case for a sufficient period of time should be made. In this way an undoubted diagnosis may be made in most cases. Yet a few diagnoses will remain to be made post-mortem. There is wide difference in the clinical course of cases on account of the suppurative process varying within the wide limits in its virulency and local destructive effects. Five types of clinical evolution have been described by French authorties. 1, a sub-acute evolution more or less distinctly divided into three stages, with an initial febril stage characteristic of septic infection; headache, vomiting and fever. This state may be confused with specific fever. It lasts a variable number of days, and is the state of suppuration. The second state is that of remission, sometimes suddenly, but more often gradually, the symptoms abate and give place to a calm which is deceptive and prolonged; and during this stage there are few or no manifest symptoms. Yet, when the abscess is in the cerrebellum, there will be some emaciation and impairment of the general health, and thorough examination would reveal some pathognomonic localizing sign. The third, or paralytic state supervenes suddenly, with or without convulsions. This may pass into profound coma, terminating fatally in a few hours, or recovery from the convulsive seizure may take the place of symptom localizing the lesion. With the onset of the third stage is generally a rising temperature. The rapidly fatal cases are usually from rupture of the abscess. The others have a more or less rapid extension of the suppuration.

See Starr, page 571, describing case of middle ear disease.

2. Evolution with severe general infection.

These cases are rapidly fatal, the abscess symptoms begin merged into those of grave general infection. High fever and delirious mania are prominent symptoms

3. Evolution with complete Latency until the final attack or coma, the patient dying suddenly or in a few hours, and an abscess that has existed usually for a long period is found at the autopsy. In some such cases death is abolutely sudden. The abscess in such cases may be found in the frontal lobe or in the outer region of the occipital lobe, and even more frequently in the right temporal lobe. In these silent cases an examination of the optic nerve head, the field of vision, and the action of the muscles of the eye has revealed the gravity of an illness which has been regarded as trivial.

4. In the fourth type of clinical evolution, the course is just like that of brain tumor.

5. The fifth type of evolution is remittent, the course being in two acts. The first is marked sometimes by headache and fever, sometimes by an attack of mania, and sometimes by acute delirium; then all quiets down and the patient seems cured, but after a few weeks or months, or even a year, there is a recurrence of symptoms which is quickly fatal. Bristow's influenza cases previously referred to, are examples of this type of evolution. In localizing the abscess the position of the injury causing, or the cranial suppuration from which it originated, have some bearing.

Unilateral spasm, paralysis, hemianopsia, and aphasia, are important symptoms in determining the exact location of the abscess. In locating temperal abscess a certain form of aphasia that has recently been observed promises to be very helpful The memory centers for hearing are located in the first and second temporal convolutions. The relative degree of development of these vary in individuals of different degrees of education. The memory centers of sight are located in the angular gyrus and calcarine fissure. The centers in these two areas are connected by association fibres passing through the white matter beneath the cortex. Deep abscess in the left temporal lobe destroys or displaces these fibers, preventing communication. If you say the word "knife," the patient can repeat it, but cannot form any notion of what it represents any more than from a word of a foreign language. If you show him a knife he can say it is what you cut with, but he cannot recall the word knife. This is called optical aphasia, or intercortical sensory aphasia. By this symptom diagnosis of abscess in the left temporal lobe was made and verified by operation. In any suspected case this symptom should be looked for, but it is useful only in locating the abscess on the left side of right handed people, and in the right side of left handed people.

Conjugate deviation of the eyes shows irritation when toward the side of the lesion, and paralysis when from the side of the lesion.

*Diagnosis.*—The diagnosis of brain abscess may be made without great difficulties in ordinary traumatic cases, for there is history of the injury and the exact location and development of a series of cerebral symptoms pointing to localized disease in the brain. Nervous symptoms coincide with the location of the wound, and the localization of the cerebral disease, and the existence of the abscess is reasonably certain. When there is no history of an injury the diagnosis is more difficult, because acute tuberculosis give the same symptom complex. Cases of brain abscess from otitis media must sometimes be distinguished from meningitis and thrombosis of the lateral sinus. The relative frequency of these conditions are not far from the same.

Poulen collected thirty-six cases of cerebral complications of ear disease. Thirteen of these were abscess, twelve were thrombosis, and eleven meningitis, there is usually a more rapid onset and progress of the symptoms than in abscess. The headache is more severe. There is hyperesthesia to sound and light and touch all over the body, these being absent in abscess, the temperature is high and rarely ever goes below normal. The pulse is rapid, irregular and intermittent. There are occasional twistings of the limbs, or slight convulsions; strabismus appears early, trismus is common, pain and rigidity of the neck are present, micro-organisms are to be found in the cerebro-spinal fluid.

In thrombosis of the lateral sinus, there will be high fever, with septic variations in range, and frequent chills. In the course of twenty-four hours the temperature may twice sink below normal and rise to 105 F. The pulse is rapid and irregular, but not intermittent. There may be tenderness, swelling and edema over the mastoid, and edema of the neck. The jugular vein may stand out as a hard, blue cord on the side of the neck.

The diagnosis of abscess arising under other conditions is never positive. The presence of a cause, and a record of cerebral symptoms that occur in a definite series of stages point strongly to presence of an abscess.

*Prognosis.*—This will depend upon the possibility of evacuating the abscess and draining it successfully. Spontaneous recovery has been recorded, but this is so rare as never to justify waiting.

Necrosis and caries of the tegmen was so great in one of two cases of tempero-sphenodal abscess as to allow them to spontaneously drain freely and successfully. This accurred just as it has done in a few cases of suppurating appendix. Star reports a collection of fifty-five cases operated. Twenty-four were after injury, twenty-four after middle ear disease, and three after typhoid. Thirty-four recovered, and twenty-one died.

In a group of sixty cases of trasmatic abscess operated, thirty-eight recovered, twenty-two died. In a collection of one hundred and ninety-six otitis abscesses operated, ninety-six recovered.

Dunch operated on nine cases and three recovered. Barnhill operated on eight cases, two of which were alive and appeared to be cured four years afterwards, and one of the others died of a recurrence four months after the operation.

One swallow doesn't make a summer, and even if death followed every operation in a *small* group of cases, who could have the temerity to question the true spirit of surgery in an effort to evacuate and drain *all* of them.

As far back as July, 1905, Korner reported ninety-two operations with fifty-seven recoveries, and twenty-nine cases of sinus thrombosis with forty-one recoveries. It is more than probable that part of the cases in which recovery does not occur, the operation had been too long delayed, or was not sufficiently exploratory.

*Treatment.* 1.—General considerations. An abscess in the brain should be dealt with just as with abscess in other parts of the body, that is, by incision and thorough drainage, and if escapulated, emucleation.

In operating, the surgeon must find out as he goes its situation, and whether acute or chronic, diffused or circumscribed, or whether there be a second one. A case of acute cerebellar abscess, opened with relief to the symptoms, died and at necropsy an old encapsuled abscess was found still further in.

When the abscess is drained, the brain tissue, which is of liquid texture, tends to fill the space, and shut off a portion of the cavity from the point of incision.

The integrity of certain parts of the brain are essential to the continuance of life, and places a limit to surgical interference in certain directions.

These general considerations do not affect the principles of treatment, but have important bearing upon the details of the operation.

2. *Operation for Brain Abscess following Local Granial Diseases.*—The operation for brain abscess should be a direct continuance of that for the removal of the continuous tract of diseased bone through which the infection spreads. This track must be followed through the bone to the interior of the skull.

If, after a mastoid operation, or Stake's operation for mecrosis of bone in the tympanum, the symptoms point clearly to abscess in the cerebellum, or tempero-spenoidal lobe, the surgeon should work his way, in the one case from the inner, or posterior wall of the antrum to the posterior surface of the petrous, or through the roof of the tympanerm.

Enough of the petrous and squama must be removed to expose the extra dural abscess, or the diseased portion of dura to which the brain abscess is attached by its stalk. By this method it is known whether the abscess has a stalk, or is isolated. This stalk is a ready-made drainage tube, and is less liable to be closed from the liquid consistence of the brain than one made by the surgeon, also infection of the meninges is far less likely, as well as hernia cerebri.

In cerebellar abscess, the stalk is attached to the dura over the sinus groove, or over the aque-ductes vestibuli, or over the internal auditory meatus. In tempero-spenoidal abscess, it is over the anterior surface of the petrous, most commonly over the tegmen.

Frontal abscess is attacked to the cranial wall of the sinus, or the anterior surface of petrous.

In deep abscess following an injury the stalk is attached over the region of the tracture. Drainage through the stalk would remove the symptoms and tendency to death, but there are cases that will require a counter opening, as in other parts of the body. To do this, remove a considerable area of bone, *open the dura* and pack with gauze until this area of the brain for incision is walled off by adhesions. This avoids diffuse encephalitis and meningitis. The area of bone removed should be in proportion to size of abscess as determined by probe through the stalk.

If respiration ceases the abscess must be evacuated in the shortest time and easiest way, and the local bone disease let alone for the time being.

Mr. Ballance, on two occasions completed the operations during the performance of artificial respiration made necessary by the first few inhalations of chloroform. Also in another, in which artificial respiration had been in progress two hours before he arrived. And he insists that neither morphia nor strychnia should be administered before the dura has been opened.

3. *Discovery nd Incision of Abscess.*—The obscess may burst when the dura is opened, and pus may be projected more than two feet. When sufficient opening through bone and dura and bone is made, palpation may show it to be immediately subcortical. Incision through the cortex may be made, with great care to avoid wounding any of the numerous vessels. The best instrument for exploration is a sharp pointed, long, narrow knife, and it should be borne in mind that the abscess is nearly always close to the bone disease giving rise to it. Clean cut wounds heal quickly, and it is easier to find the abscess with the knife.

The trocar and canula have missed the abscess, or passed through without tapping it, or struck the capsule and failed to penetrate it. Dupuytren, in one of his lectures, says: "In certain cases of deeply seated fluid collections, we must incise the dura and arachnoid and brain itself, and by this bold proceeding, patients have been saved." In the same lecture further on he continued: "Relying also on the success of J. L. Petit, Boyer concurs in the advice of Quesnay, and does not fear to plunge the bistoury quite deeply into the very substance of the brain in order to evacuate traumatic effusions, and it has fallen to my lot several times to do so with success." Fifty years later an English surgeon wrote: "There are few surgeons who would have the hardihood of Dupuytren, who plunged a bistoury in the substance of the brain, and thus luckily relieved the patient of an abscess in this situation." In his account of this, Dupuytren says: "I incised the dura, nothing came out; I thrust the bistoury cautiously into the brain and there welled up immediately a flood of pus! That very night all the symptoms disappeared, and the patient recovered." If the knife failed to find the abscess, it is quite easy to find it with the finger, as a tense, abnormal swelling, which may be opened by the knife guided and guarded by the finger. A second obscess may be mistaken for the teutorium.

In two cerebellar cases, one abscess in one and two in the other, were drained, and yet both patients died from an unopened, oyster shaped abscess just beneath the cortex of the upper surface.

*Progress of the Case*.—The course of brain abscess is variable. The earlier the operation is done the greater the chance of recovery. There should never be delay after diagnosis is made, and the operation should always be done unless the patient is actually moribund.

The operation has been done during artificial respiration and the patient recovers.

After the operation the patient may rapidly convalesce, or present symptoms which will tax to the utmost the resources of the surgeon. A voracious appetite is a favorable sign.

Less than a generation ago but few sergeons ever attempted to operate for brain abscess. During the last twenty years recoveries achieved make the future of this field bright with promise.

